# Cardiac Rehabilitation in Advanced aGE after PCI for acute coronary syndromes: predictors of exercise capacity improvement in the CR-AGE ACS study

**DOI:** 10.1007/s40520-022-02130-y

**Published:** 2022-04-22

**Authors:** Samuele Baldasseroni, Maria Vittoria Silverii, Alessandra Pratesi, Costanza Burgisser, Francesco Orso, Giulia Lucarelli, Giada Turrin, Andrea Ungar, Niccolò Marchionni, Francesco Fattirolli

**Affiliations:** 1 Department of Experimental and Clinical Medicine, University of Florence, Florence, Italy; 2 Cardiac Rehabilitation Unit, Azienda Ospedaliero-Universitaria Careggi, Largo Brambilla 3; 50134, Florence, Italy

**Keywords:** Elderly, Cardiac rehabilitation, Acute coronary syndrome, Exercise capacity

## Abstract

**Background:**

The positive effect of cardiac rehabilitation (CR) on outcomes after acute coronary syndromes (ACS) is established. Nevertheless, enrollment rates into CR programs remain low, although ACS carry a high risk of functional decline particularly in the elderly.

**Aim:**

We aimed to determine if a multidisciplinary CR improves exercise capacity in an older population discharged after ACS systematically treated with PCI.

**Methods:**

CR-AGE ACS is a prospective, single-center, cohort study. All patients aged 75+ years consecutively referred to Cardiac Rehabilitation outpatient Unit at Careggi University Hospital, were screened for eligibility. Moderate/severe cognitive impairment, disability in 2+ basic activities of daily living, musculoskeletal diseases, contraindication to Cardiopulmonary Exercise Test, and diseases with an expected survival < 6 months, were exclusion criteria. Participants attended a CR program, based on 5-day-per-week aerobic training sessions for 4 weeks.

**Results:**

We enrolled 253 post-ACS patients with a mean age 80.6 ± 4.4 years. After CR, 136 (56.2%) 77 (31.3%) patients obtained, respectively, at least a moderate (∆+5%) or an optimal (∆+15%) increase in VO_2_peak. Baseline VO_2_peak (− 1 ml/kg/min: OR 1.18; 95% CI 1.09–1.28), the number of training sessions (+1 session: OR 1.07; 95% CI 1.01–1.15), and mild-to-moderate baseline disability (yes vs. no: OR 0.22; 95% CI 0.01–0.57) were the predictors of VO_2_peak changes.

**Conclusions:**

A CR program started early after discharge from ACS produces a significant increase in exercise capacity in very old patients with mild-to-moderate post-acute physical impairment. Baseline VO_2_peak, the number of training sessions, and the level of baseline disability are the independent predictors of improvement.

## Introduction

The positive effects of cardiac rehabilitation (CR) on cardiovascular mortality, re-hospitalizations, and quality of life have been established [1] and current guidelines strongly suggest an early planning of interventional secondary prevention after acute coronary syndromes (ACS) [2, 3]. Few studies that enrolled elderly patients demonstrated a significant improvement in exercise capacity with CR also in this age cohort [4]. Despite proven benefits, CR enrollment rates remain remarkably low in the elderly in general, and in older women in particular [5]. Indeed, referral to CR at hospital discharge is even lower in patients treated with percutaneous coronary intervention (PCI) for ACS than after cardiac surgery [6], probably reflecting physicians’ perception that functional recovery is less stringently needed after ACS than after cardiac surgery [7]. Conversely, discharge from hospital where PCI has been performed [8] and where facilities for CR program are present [9], are strong positive predictors of referral to CR. The physician perception of less functional impact of ACS than cardiac surgery in the elderly [10] is contradicted by observations of about 30% of older patients having a remarkable functional decline six months after ACS, with an increased risk of long-term adverse events [10]. The need for multidisciplinary therapeutic interventions aimed at improving exercise capacity and reducing the risk of physical and also cognitive disability is becoming a priority in older cardiovascular patients [11]. Among different indexes of functional capacity, the maximal oxygen consumption (VO_2_peak, ml/kg/min) measured at cardiopulmonary exercise test (CPET) remains a gold standard [11, 12].

With the aim of investigating the effect of CR in older patients [13], we have conducted the Cardiac Rehabilitation in Advanced aGE (CR-AGE) single-center, prospective study, which recently identified the subset of patients benefiting the most from CR in terms of improved exercise capacity [14].

Based on these premises, the present study was aimed at identifying the independent predictors of improvement in VO_2_peak at the end of a multidisciplinary CR program in an older population discharged after ACS systematically treated with PCI.

## Methods

Over the last 10 years, 1580 patients older than 75 years have been discharged from our hospital after an ACS treated with PCI. In the absence of standardized procedure to refer eligible patients to CR, the referral decision was made by acute-care physicians and, therefore, was based only on physician’s clinical judgment and acknowledgement of potential CR benefits.

**Fig. 1:**
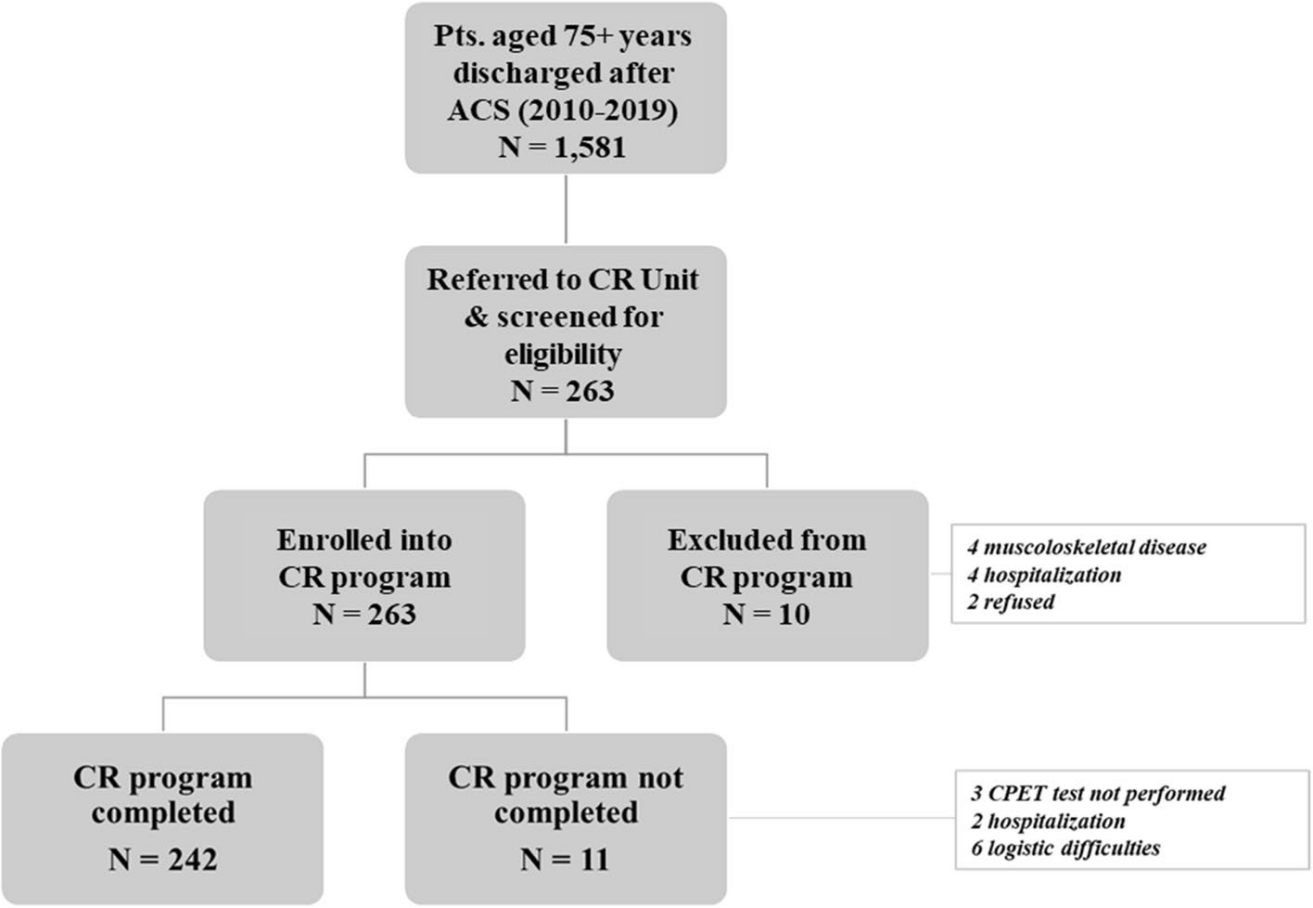
Study flowchart

The CR-AGE protocol, including the description of the methods and data collected, has been detailed elsewhere [14] and can be summarized as follows. The program consists of 5-day-per-week sessions of aerobic exercise for 4 weeks, at an intensity corresponding to 60–70% of VO_2_peak measured in a baseline with a symptom-limited cardiopulmonary stress test as a measure of maximal functional capacity at the entry. This program duration reflects the length of CR usually provided by the Italian national healthcare system. Each session consists of 30 minutes of either biking or calisthenics on alternate days, with an expert physiotherapist supervising activities through telemetric ECG and non-invasive arterial blood pressure monitoring [14]. Based on the same protocol, in the present study we screened for eligibility for CR all patients aged 75+ years consecutively referred to our CR Unit (Fig. 1). Accordingly to the CRAGE protocol, moderate-to-severe cognitive impairment (Mini-Mental State Examination score < 18) [15], disability in 2+ basic activities of daily living (BADL) [16], ejection fraction equal or less 35%, musculoskeletal diseases or other absolute contraindication to CPET, and diseases with an expected survival < 6 months, were taken as exclusion criteria [14, 17].

All patients were evaluated through a comprehensive geriatric assessment process, which included the definition of chronic comorbidity burden [18], independence in activities of daily living, and psycho-emotional [19] and socio-economic profile. Loss of only one BADL and/or one or more instrumental ADL (IADL, 20) was taken to indicate mild-to moderate disability, but did not cause patient’s exclusion.

### Functional evaluation

Changes in aerobic capacity at the end of the 4-week physical training (see below) was the main study outcome measure. Aerobic capacity was expressed as the VO_2_peak resulting from breath-to-breath analysis (CPX Medical Graphics system) during a symptom-limited CPET on cycle ergometer (Esaote Biomedica Formula). All participants screened for participation in study were prescribed the above described [14], 4-week training program.

### CR program

The CR program was individually customized and the work load of exercise sessions, consisting of 30-minute biking or calisthenics on alternate days, was changed weekly according to the Borg rating of perceived exertion scale [21]. The calisthenics program consisted of a warm-up period followed by eight 2-minute exercises, each followed by 1-minute rest, and of stretching of the trunk muscles aimed at improving strength and flexibility and, hence, autonomy in common daily activities. A progressive increase in muscle resistance was reached by applying ankle or wristbands of increasing weight (0.5-1 kg), again based on the Borg rating of perceived exertion scale, which was re-evaluated weekly. As already reported, sessions were supervised by an expert physiotherapist, with telemetric ECG and non-invasive arterial pressure monitoring [22]. The study has been approved by the local ethics committee and has been conducted in accordance with the Declaration of Helsinki.

### Statistical analysis

Data were analyzed using the SPSS 25.0 statistical package (SPSS, Inc., Chicago, IL). Continuous and categorical variables are reported respectively as mean ± standard error, or as N and percent. The univariable associations of demographic, clinical, and echocardiographic variables with indexes of exercise performance derived from CPET at the end of CR, were tested using Student’s *t* test and chi-square tests, as appropriate. For this analysis, according to the results of previous studies [14], thresholds indicating at least a moderate or an optimal increase in VO_2_peak from baseline to the end of CR program, were set respectively at > +5% and ≥ +15%. Variables significantly associated with either a moderate or an optimal final increase in VO_2_peak at univariable analysis, were entered into multivariable logistic models (with stepwise backward deletion), to identify the independent predictors of the two thresholds of exercise capacity increase. A *p* value < 0.05 was considered statistically significant.

## Results

We consecutively enrolled 253 patients (Fig. 1) aged ≥ 75 years (mean age 80.6±4.4 years, range 75–94; 116 males, 65.6%) who had been treated with PCI during an ACS (156 STEMI, 61.7%; 96 NSTEMI/unstable angina, 37.9 %) (Table 1). Of these, 93 (36.8%) had a single-, 84 (33.2%) a two-, and 71 (31.1%) a three-vessel disease; 151 (59.3%) had had a complete coronary revascularization, and 177 (70.0%) had received at least one drug-eluting stent. The average intervals from ACS and from hospital discharge to enrollment were 19 ± 11 and 11 ± 10 days, respectively.

**Table 1:** Demographic and clinical characteristics of the study population

	*N* = 253
Age (years)	80.6 ± 4.4
Male gender	65.6 (166)
BMI (Kg/m^2^)	26.3 ± 0.2
Hypertension	73.5 (186)
Diabetes	24.1 (61)
Dyslipidemia	46.6 (118)
Current smoking	21.7 (55)
COPD	9.1 (23)
BADL preserved	5.7 ± 0.4
IADL preserved	7.1 ± 0.1
Mild-to-moderate disability	9.5 (24)
Charlson comorbidity index score	5.8 ± 1.0
MMSE score	27.7 ± 0.1
15-item GDS	3.5 ± 0.2
RAAS inhibitors	91.3 (231)
Beta-blockers	86.6 (219)
Statins	94.5 (238)
NSTEMI-UA	37.9 (96)
STEMI	61.7 (156)
Complete revascularization	59.3 (151)
LVEF (%)	53.7 ± 0.6
Baseline VO_2_peak (ml/kg/min)	13.4 ± 0.2

Continuous variables: mean±SE; categorical variables: % (*n*)

*BMI* body mass index, *COPD* chronic obstructive pulmonary disease, *BADL/IADL* basic/instrumental activities of daily living, *MMSE* Mini-Mental State Examination, *GDS* Geriatric Depression Scale, *RAAS* Renin-Angiotensin-Aldosterone System, *NSTEMI-UA* non-S-T segment elevation myocardial infarction/unstable angina, *STEMI S-T* segment elevation myocardial infarction, *LVEF* left ventricular ejection fraction, *VO*_*2*_*peak* maximal oxygen consumption

In accordance with the exclusion criteria, 80.5% of patients were independent in 6/6 BADL and only 23.8% had limitations in two or more IADL, while the average cognitive and psycho-emotional profiles were good, as indicated by a MMSE score of 27.6 ± 3.2 and a 15-item GDS score of 3.5 ± 3.0. A Charlson comorbidity index of 5.7 ± 1.6 indicated a moderate average burden of non-cardiovascular chronic comorbidity. Overall, the prescription rate of guidelines-recommended therapies after ACS at CR entry was very satisfying (Table 1), particularly if we consider the high mean age of the study population. Patients participated in 13±4 physical exercise sessions as previously described, under physiotherapist’s supervision.

**Fig. 2:**
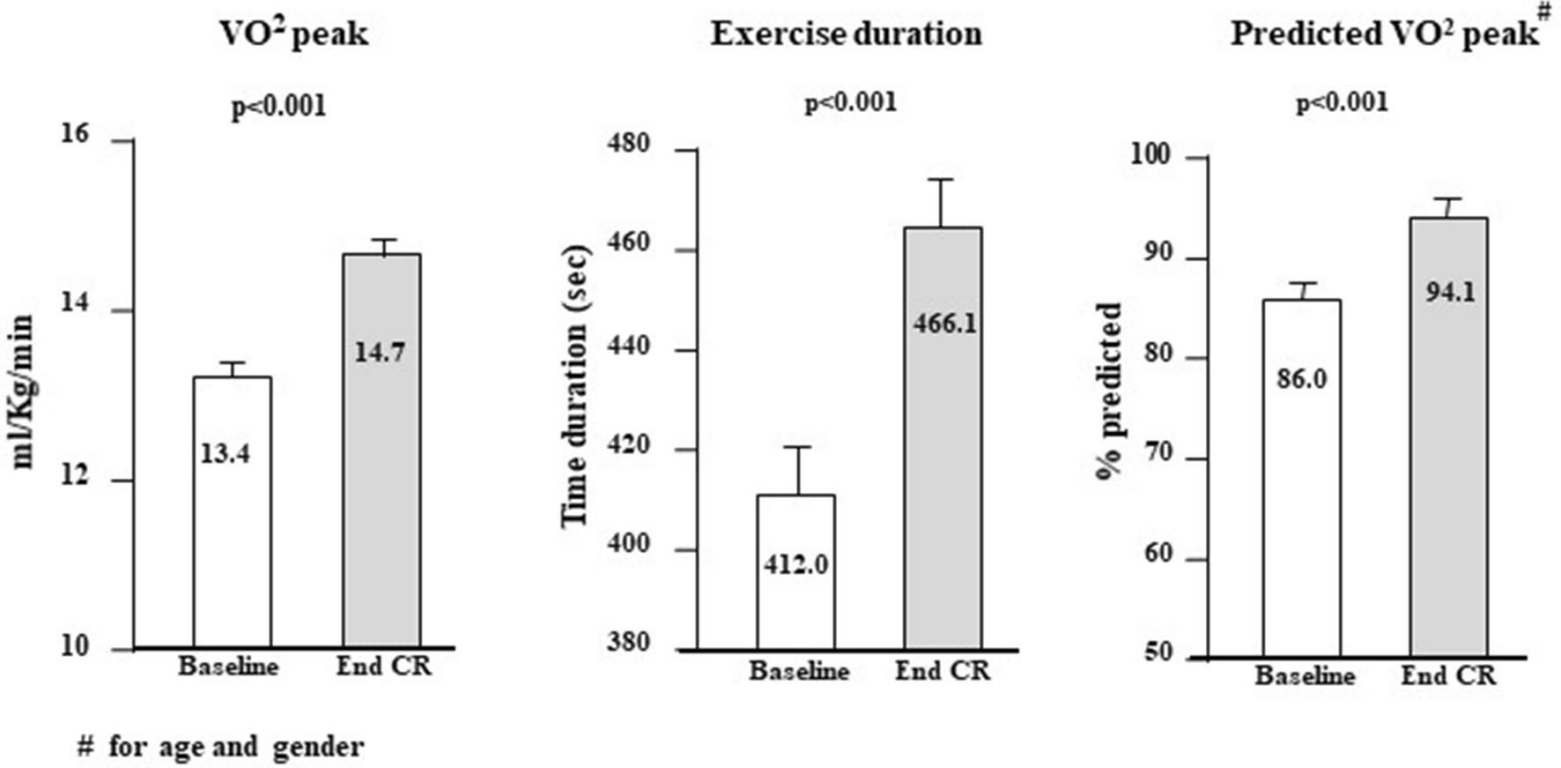
Changes in maximal oxygen consumption (VO_2_peak ml/kg/min), exercise duration (second) and percent VO_2_peak predicted for age and gender, from baseline to end of CR

Of 253 patients enrolled, 242 (94.2%) completed the whole CR program and underwent a final CPET before discharge (Fig. 1), whereas 11 patients initially enrolled could not attend the CR program due to lacking familial support or logistic difficulties. VO_2_peak and exercise duration remarkably and significantly improved from baseline to final evaluation (Fig. 2). When changes in VO_2_peak from baseline to the end of CR program were analyzed according to the two pre-defined thresholds, 136 (56.2%) and 77 (31.3%) resulted to have reached an increase in VO_2_peak > 5% or ≥ 15%, respectively. At univariable analysis, attainment of the lower threshold was significantly associated with number of IADL lost, presence of mild-to-moderate disability, baseline VO_2_peak, and the number of training sessions attended, while attainment of the upper threshold was associated with MMSE score, baseline VO_2_peak and the number of training sessions (Table 2).

**Table 2:** Association of clinical variables with either a “Moderate” or an “Optimal” increase in VO2peak from baseline to the end of CR program *(N* = 242*)*

	“Moderate” improvement: final VO_2_peak Δ > +5% from baseline			“Optimal” improvement: final VO_2_peak Δ ≥ +15% from baseline		
	No *N* = 107	Yes *N* = 135	*p* value	No *N* = 165	Yes *N* = 77	*p* value
Age (years)	80.9 ± 0.4	80.4 ± 0.4	0.401	80.6 ± 0,5	80.6 ± 0.5	0.670
Male gender	67.3 (72)	64.8 (86)	0.561	67.7 (110)	68.3 (48)	0.510
BMI (Kg/m^2^)	26.0 ± 0.4	26.6 ± 0.3	0.254	26.1 ± 0.3	26.8 ± 0.4	0.205
Hypertension	74.8 (80)	71.1 (96)	0.526	71.5 (118)	75.3 (58)	0.535
Diabetes	22.4 (24)	25.2 (34)	0.618	24.8 (41)	22.1 (17)	0.638
Dyslipidemia	45.8 (49)	45.9 (62)	0.984	44.2 (73)	49.4 (38)	0.458
Current smoking	21.5 (23)	23.7 (32)	0.684	24.8 (41)	18.2 (14)	0.249
COPD	12.1 (13)	6.7 (9)	0.141	10.3 (17)	6.5 (5)	0.337
BADL preserved	5.8 ± 0.1	5.8 ± 0.0	0.842	5.8 ± 0.0	5.8 ± 0.1	0.318
IADL preserved	**6.9 ± 0.2**	**7.4 ± 0.1**	**0.003**	7.1 ± 0.1	7.4 ± 0.1	0.115
Mild-to-moderate disability	**16.0 (17)**	**5.2 (7)**	**0.005**	11.6 (19)	6.5 (5)	0.218
Charlson Comorbidity Index score	5.7 ± 0.2	5.8± 0.1	0.641	5.7 ± 0.1	5.7 ± 0.2	0.975
MMSE score	27.6 ± 0.2	28.0 ± 0.2	0.373	**27.5 ± 0.2**	**28.2 ± 0.2**	**0.019**
15-item GDS	3.5 ± 0.3	3.3 ± 0.2	0.667	3.5 ± 0.2	3.2 ± 0.3	0.415
eGFR (ml/min/1.73 m^2^)	64.3 ± 1.7	61.0 ± 1.4	0.134	62.5 ± 1.4	62.2 ± 1.8	0.893
Hb (g/dL)	12.5 ± 0.1	12.8 ± 0.1	0.183	12.6 ± 0.1	12.7 ± 0.2	0.931
Total protein (mg/dL)	6.9 ± 0.1	6.5 ± 0.0	0.965	6.9 ± 0.1	6.9 ± 0.0	0.895
RAAS inhibitors	90.7 (97)	91.9 (124)	0.742	92.7(153)	88.3 (68)	0.256
Beta-blockers	82.2 (88)	88.9 (120)	0.140	84.8(146)	88.3 (68)	0.470
Statins	93.5 (100)	95.6 (129)	0.472	95.2(157)	93.5 (72)	0.597
NSTEMI-UA	43.9 (47)	32.8 (44)	0.078	40.6 (67)	31.6 (24)	0.179
STEMI	56.1 (60)	67.2 (90)		59.4 (98)	68.4 (53)	
Complete revascularization	60.0 (63)	61.7 (82)	0.525	62.9 (102)	58.7 (44)	0.663
LVEF (%)	53.7 ± 0.9	53.4 ± 0.8	0.908	53.5 ± 0.8	53.9 ± 1.0	0.786
Baseline VO_2_peak (ml/kg/min)	**14.7 ± 0.4**	**12.6 ± 0.3**	**0.001**	**14.2 ± 0.3**	**12.0 ± 0.4**	**0.001**
N. training sessions	**12.6 ± 0.4**	**13.8 ± 0.4**	**0.022**	**12.8 ± 0.3**	**14.3 ± 0.5**	**0.011**

*eGFR* estimate glomerular filtration rate, *BMI* body mass index, *COPD* chronic obstructive pulmonary disease, *BADL/IADL* basic/instrumental activities of daily living, *MMSE* Mini-Mental State Examination, *GDS* Geriatric Depression Scale, *RAAS* Renin-Angiotensin-Aldosterone System, *NSTEMI-UA* non-S-T segment elevation myocardial infarction/unstable angina, *STEMI S-T* segment elevation myocardial infarction, *LVEF* left ventricular ejection fraction, *VO*_*2*_*peak* maximal oxygen consumption

Bold characters highlight statistical significance

At multivariable analysis, baseline VO_2_peak and the number of training sessions were the strongest independent predictors of both thresholds of VO_2_peak increase at the end of CR program, whereas the number of IADL preserved and the presence of mild-to-moderate disability were retained in the model as predictors of the lower threshold only, and the MMSE score was marginally associated with the upper threshold only (Table 3).

**Table 3:** Independent predictors of a “Moderate” or an “Optimal” increase in VO_2_peak from baseline to the end of CR program (multivariable logistic regression analysis)

Variable	“Moderate” improvement: final VO2peak Δ > +5% from baseline (*R*^2^ = 0.18)		“Optimal” improvement: final VO_2_peak Δ ≥ +15% from baseline (*R*^2^ = 0.24)	
	OR (95% CI)	*p* value	OR (95% CI)	*p* value
Baseline VO_2_peak (for each ml/kg/min decrease)	1.18 (1.09–1.28)	< 0.001	1.18 (1.08–1.17)	< 0.001
N. of training sessions (for unitary increase)	1.07 (1.01–1.15)	0.029	1.08 (1.01–	0.037
Mild-to-moderate disability (yes vs. no)	0.22 (0.08–0.57)	0.002	……………….	……
N. of baseline IADL preserved (for unitary decrease)	1.21 (0.83–1.76)	0.316	………………	……
Baseline MMSE score (for unitary increase)	……………….	………	1.15 (0.99–1.34)	0.069

*BMI* body mass index, *COPD* chronic obstructive pulmonary disease, *BADL/IADL* basic/instrumental activities of daily living, *MMSE* Mini-Mental State Examination, *GDS* Geriatric Depression Scale, *RAAS* Renin-Angiotensin-Aldosterone System, *NSTEMI-UA* non-S-T segment elevation myocardial infarction/unstable angina, *STEMI S-T* segment elevation myocardial infarction, *LVEF* left ventricular ejection fraction, *VO*_*2*_*peak* maximal oxygen consumption

## Discussion

The first finding of our study to be highlighted is the advanced mean age (greater than 80 years) of enrolled patients, who had all been treated with an early invasive strategy during an ACS. Such an aggressive strategy, together with the adopted exclusion criteria, likely resulted in a well preserved average clinical and functional profile that was associated with a high rate (94.2%) of patients completing the multidisciplinary 4-week outpatient CR program. Moreover, we confirmed the effectiveness of CR in improving the global exercise capacity also in such a geriatric cohort.

As known, physical-function maintenance is a crucial target for clinical interventions in the elderly, and exercise training is a key tool to improve and promote secondary prevention in coronary artery disease [22]. Nevertheless, an increase in physical exercise performance is of crucial importance for maintaining the independence in activities of daily living, and is associated with an improvement in health related quality of life [11]. In the elderly, this positive effect should be obtained soon [23] after ACS, to prevent the negative effects related to hospital stay, associated with deconditioning caused by muscle mass and strength loss that occurs even after a short length of stay [24]. In addition, an early CR program can contribute to rebuild patient’s confidence with physical activities [11] . Our structured CR programs are run by trained physiotherapists, who are in charge of coordinating tailored on-site exercise programs and, at the same time, of educating patients to continue the same programs at home. [14].

Despite all aforementioned positive results and derived clinical practice guidelines recommendations [5, 17], enrollment in CR still remains poor [7, 9] with physicians’ decision still resulting as the main barrier to enrollment [7]: in fact, many potentially eligible patients, particularly older adults, are not referred as a consequence of multiple factors such as health conditions, hospital policies, but also physicians’ unjustified perception of limited usefulness [8, 23]. Hopefully, in this perspective, our findings may contribute to contrast this phenomenon so difficult to explain in the light of the above described solid evidence in favor of CR referral, particularly in elderly patients.

Indeed, in a large US population [25], age resulted to be per se a significant barrier to patients referral to a CR program after PCI: in fact, every 5 years of increasing age caused a 2% reduction in the probability of being referred to CR, even after adjusting for cardiovascular risk profile, comorbidities, hospital facilities, type of insurance and ethnicity. In our study, only 17% of older patients discharged from hospital over 10 years have been referred to CR. These data are consistent with the literature, which reports that older adults are 1.5–2 times less likely to participate in CR, with a referral rate dropping to only 13% in those aged 80 + years [26, 27].

A further valuable finding of the present study is the achievement of a significant increase in VO_2_peak with multidisciplinary CR program in a large proportion of our study population. Moreover, we have confirmed in a large and more clinically uniform population of older patients with ischemic heart disease, the findings of our previous CR-AGE study [14], that greatest gains in exercise capacity occur in those who have the lowest baseline physical performance. In fact, we observed that for each ml/kg/min lower baseline VO_2_peak, there was an independent 18% increased probability to obtain a functionally remarkable increase in exercise tolerance. This finding reinforces the positive impact of an early referral of older patients to a CR program that can largely and rapidly improve the aerobic capacity particularly in those patients with the most impaired physical performance at hospital discharge [14]. Recently, it has been demonstrated, in older post-MI patients, that frailty (defined as a slow gait speed) and non‐participation to CR, were independent predictors of 1-year mortality and incident disability, and we know to what extent impairment in physical performance is a central domain of frailty and, hence, of further loss of physical function and incident disability [28, 29].

Indeed, it is well known that a decreased VO_2_peak, together with comorbidities and sedentariness, contribute to feed a vicious circle made of worsening cardiac disease and progressive loss of physical functioning, with a consequent high risk of incident BADL/IADL disability [12]. Therefore, it is not surprising that, in functionally frail individuals, an even small increase in VO_2_peak can produce a substantial reduction in all-cause and cardiovascular mortality [30]. However, though the individual response to CR is largely variable, exercise programs proved to be safe and to improve the functional capacity and reduce myocardial work to a similar extent in older and younger coronary patients [31]. The variability in CR beneficial effects has been highlighted by authors of the SAINTEX-CAD study, who demonstrated that as many as one-third of patients did not obtain a significant improvement in exercise capacity [32], despite high participation in the CR program. In that study, older age, elective PCI, higher baseline VO_2_peak, and oxygen uptake efficiency slope, as well as lower training intensity and baseline energy expenditure, but not the number of training sessions, were the predictors of non-response to training. In our study, the number of training sessions turned out to be a significant positive predictor of response to CR expressed in terms of a moderate or an optimal VO_2_peak increase from baseline to the end of CR. We hypothesize that this difference might be related to a remarkable age difference between the two study populations (middle-age patients in SAINTEX-CAD vs. very old participants in our study) and, most importantly, to a different duration of the CR program (12 vs. 4 weeks). Therefore, because of the high number (N=36) of training sessions in both physical exercise groups, a ceiling effect, above which a further increase in VO_2_peak cannot be obtained, might have occurred in SAINTEX study. Whether increasing the duration of training sessions of the CR program can improve the cardiopulmonary exercise capacity, as well as the optimal modalities to supervise the exercise capacity of patients after an ACS in the follow-up period, remains to be further assessed in older patients.

It is interesting to note that, in the present CR-AGE ACS study, neither the extension of coronary disease, nor the type of myocardial infarction (NSTEMI vs. STEMI), nor the completeness of myocardial revascularization, were predictors of aerobic capacity improvement. As a side note, in our population we observed a higher prevalence of STEMI, whereas NSTEMI/UA are usually more prevalent in older patients, probably because we selected patients treated in any case with PCI.

We must underline how we cannot debate about the predictive role of reduced ejection fraction and/or overt heart failure in our study population. The importance of physical activity or structured cardiac rehabilitation in the field of heart failure caused by ischemic or no ischemic diseases is clearly established in term of its effect on outcomes (33 REF-Cacciatore). The reasons of this lack is related to different reasons: first, in the CRAGE protocol [14] ejection fraction equal or less 35% was an exclusion criteria; consequently those patients with HFrEF could not be enrolled. Second, as reported in the method section we enrolled patients early from discharge after acute coronary syndrome. This fact limited greatly to refer us patients with history of heart failure or incident acute heart failure soon after an acute coronary syndrome treated with PCI, unless to enroll patients clinically unstable but, of course, not eligible at this time for cardiac rehabilitation program (34 JAMA. 2009;301:1439-1450).

Finally, an interesting finding regards the influence of baseline functional independency on the improvement of the exercise capacity at the end of CR. We found that even a mild-to-moderate decline in functional independency (defined as a loss of 1+ BADL or 2+ IADL) can negatively affect the probability to reach an increase ≥ 5% in VO_2_peak from baseline. Older patients often become sedentary after acute cardiac events, due to physical deconditioning after an even short hospitalization [35] and because of reactive depressive symptoms that negatively impact on functional recovery [36]. Such a sedentariness may lead to further global functional decline in the early post-acute clinical course [37]. In this scenario, a baseline comprehensive geriatric assessment may play a crucial role in precisely planning an individually tailored CR program in the elderly. This view is further supported by our finding that even a modest decrease of a substantially normal cognitive profile can negatively impact the maximally attainable cardiopulmonary fitness. Although this finding is slightly below the threshold of statistical significance, we believe that it is consistent with the general evidence of a close association of cognitive function with cardiorespiratory fitness [38].

Study limitations can be summarized as follows: The first study limitation is its observational nature. Second limitation is the absence of a control group even if the aim of the present study was not to compare the effect of CR vs usual care, but it was to identify the independent predictors of changes in physical performance in this real world and homogenous population of older patients recovering from an ACS; all treated with early invasive strategy and PCI. A third limitation is the relatively short duration of CR, as 4 weeks may be regarded as insufficient to maximize the increase in VO_2_peak, especially in older adults. However, this duration reflects the routine length of rehabilitation provided by the Italian national healthcare system and, therefore, our study provides information that are relevant in the perspective of real-world rehabilitation practice in Italy. Furthermore, the availability of only two measurements (at baseline and 4 weeks later) cannot exclude the influence of a regression to the mean effect, but the average improvement observed in VO_2_peak is close to that obtained in similarly old population enrolled in other studies [14]. Finally, a selection bias represented by the exclusion of individuals with more marked cognitive decline or disability and patients affected by any clinical phenotype of heart failure, might limit, together with the fact that CR-AGE ACS study represents a single-center experience, the generalizability of these findings to the broader spectrum of older adults routinely hospitalized for ACS with or without overt HF, also because the presence of some of these conditions generally produces a more conservative approach to ACS treatment strategy or induces toward a later referral to CR program.

## Conclusions

Despite the aforementioned limitations, the CR-AGE ACS study demonstrated that a multidisciplinary, structured CR program started early after discharge in very old patients who underwent PCI for ACS, produces remarkable and clinically valuable improvements in exercise capacity in the majority of them. These results confirm that older adults with mild-to-moderate post-acute physical impairment are ideal candidates for CR program to promote their rapid functional recovery although in our study population were not included severe frail or comorbid older patients that obviously limits the widespread generalization of the finding. Our next aim will be to verify if the improvement in exercise capacity obtained during CR program is maintained in the follow-up period; because this confirmation undoubtedly will reinforce greatly the clinical message.
